# Mitotic counts in breast cancer should be standardized with a uniform sample area

**DOI:** 10.1186/s12938-016-0301-z

**Published:** 2017-02-16

**Authors:** Michael Bonert, Angela J. Tate

**Affiliations:** 1 St. Joseph’s Healthcare Hamilton, McMaster University, Hamilton, ON Canada; 2 Eastern Health and Memorial University of Newfoundland, St. John’s, NL Canada

**Keywords:** HPF, Mitotic counting, Breast cancer, Sampling, Cancer grading, Reproducibility, Simulation

## Abstract

**Background:**

Mitotic rate is routinely assessed in breast cancer cases and based on the assessment of 10 high power fields (HPF), a non-standard sample area, as per the College of American Pathologists cancer checklist. The effect of sample area variation has not been assessed.

**Methods:**

A computer model making use of the binomial distribution was developed to calculate the misclassification rate in 1,000,000 simulated breast specimens using the extremes of field diameter (FD) and mitotic density cutoffs (3 and 8 mitoses/mm^2^), and for a sample area of 5 mm^2^. Mitotic counts were assumed to be a random sampling problem using a mitotic rate distribution derived from an experimental study (range 0–16.4 mitoses/mm^2^). The cellular density was 2500 cell/mm^2^.

**Results:**

For the smallest microscopes (FD = 0.40 mm, area 1.26 mm^2^) 16% of cases were misclassified, compared to 9% of the largest (FD 0.69 mm, area 3.74 mm^2^), versus 8% for 5 mm^2^. An excess of 27% of score 2 cases were misclassified as 1 or 3 for the lower FD.

**Conclusion:**

Mitotic scores based on ten HPFs of a small field area microscope are less reliable measures of the mitotic density than in a bigger field area microscope; therefore, the sample area should be standardized. When mitotic counts are close to the cut-offs the score is less reproducible. These cases could benefit from using larger sample areas. A measure of mitotic density variation due to sampling may assist in the interpretation of the mitotic score.

**Electronic supplementary material:**

The online version of this article (doi:10.1186/s12938-016-0301-z) contains supplementary material, which is available to authorized users.

## Background

Tumour growth rate is a prognostic marker and can be evaluated by its correlate at the cellular level: mitoses. Thus, mitotic counts are used in a wide number of neoplasms to predict prognosis, and highly proliferative neoplasms (with many mitoses) usually have a worse prognosis. Mitotic counts are performed by a pathologist, counting mitotic figures at a high magnification. As mitotic figures are rare in relation to the number of cells, 10 high power fields (HPF) of view are typically examined. As cellularity is time consuming to quantify, mitoses/area is often used instead of mitoses/cell.

Considered as a sampling problem, mitotic counting is, typically biased in a number of ways: (1) many pathologists do not start the count until they have found one mitosis, (2) pathologists count mitoses in the area of the tumour they consider to be the most mitotically active (usually the most poorly differentiated portion). The former introduces a systematic bias that consistently skews the results in the direction towards a higher mitotic score. The later factor is not a significant factor if one frames the problem as an assessment of the poorly differentiated region of the tumour (as opposed to the tumour as a whole).

In breast pathology, mitotic counts are a part of the Nottingham score and have been demonstrated to be a histomorphologic predictor of outcome. The procedure for counting mitoses was laid-out in the original paper that described the scoring system [1], and has subsequently been clarified in the CAP protocol, where it states it should be done on the “most mitotically active area”. The system recognizes that mitotic rate per area is a strong predictor and essentially standardizes the cut-points (3 and 8 mitoses/mm^2^) to two separate mitotic rates (mitoses/area), creating a three tier system. The system pseudo-standardizes the sample area to 10 HPF, where one HPF is the field area seen with the 40x objective and dependent on the field diameter of the microscope.

Mitotic counting in breast pathology has been considered a sampling problem and it has been studied experimentally and modelled mathematically [3]. Experimentally assessing the reproducibility of mitotic counts rigorously is an onerous proposition, and it would be prohibitively expensive to do a large study from which the misclassification errors due to sampling can be accurately assessed. The reproducibility of mitotic counts in the context of breast pathology is “low” [3]; thus, small sample sizes (< 50 cases) are not sufficient. Unselected breast cancer cases are usually mitotic score 1, and this makes a study of misclassification more onerous, as the effect size is smaller than it would be if the cases are equally distributed among the different scores.

A computer simulation of this problem is an elegant solution as it can avoid the onerous labour and control for many confounders. Populations of simulated specimens can be randomly sampled, and compared to the true mitotic rates. Such a gold standard comparison is not possible using glass slides and pathologists. This can be done millions of times to determine the distribution of correct and incorrectly classified simulated specimens, and is a powerful tool for demonstrating the effect of sampling 10 HPFs from microscopes with different field areas.

The area of a HPF measured in mm^2^ may vary considerably from microscope to microscope. Using the smallest and largest field diameters from the table in the College of American Pathologists (CAP) checklist for Invasive Breast Cancer [2], a microscope with a field diameter of 0.40 mm has a HPF area of 0.13 mm^2^, while one with a field diameter of 0.69 mm has a HPF area of 0.37 mm^2^. This is almost a three-fold difference in the area sampled in one HPF.

HPF is a tenuous pseudo-standard and, unfortunately, this measure is widely used throughout pathology, from eosinophilic esophagitis [4] to gastrointestinal stromal tumours [5], and breast cancer [6] to mention a few. It has been recognized that the HPF, an often used measure of area, has no uniform definition and is a significant and under recognized source of variability that can lead to misclassification affecting reproducibility. As a result, there has been a gradual move toward establishing standardized sample areas, e.g. 5 mm^2^ for gastrointestinal stromal tumour [5]; however, it stubbornly persists in many areas of pathology.

Sampling problems, similar to mitotic counting, are all around us, and differences in sample size significantly effect predictions. A public opinion survey with 1000 individuals is more representative of the population than one with 500 individuals. This paper will show the same applies for the sample area, and will demonstrate how large the effect of sample size is in the context of breast cancer mitotic scoring.

This paper will rigorously assess the impact of the sample area on the mitotic score in the three-tier system used in breast pathology and it will demonstrate that a standardized sample area is essential.

## Methods

An in silico model was developed based on the binomial distribution [7], using the software GNU Octave (www.gnu.org/software/octave/). The model assumes mitotic counting is a sampling problem. It assessed the classification and misclassification rates of 1,000,000 simulated breast specimens, using the sample areas for the extremes of the field diameter range (FD = 0.40 mm, 10 HPF = 1.26 mm^2^, FD = 0.69 mm, 10 HPF = 3.74 mm^2^) in the College of American Pathologists (CAP) checklist [6], as well as the areas of 5.00 mm^2^, which would be equivalent to 40 HPF at a FD 0.40 mm, or almost 14HPF for FD 0.69 mm.

Each simulated breast specimen was assigned a true mitotic density, based on an experimentally determined distribution from Meyers et al. The mitotic rate in the population ranged from 0 to 16.4 mitoses/mm^2^ and is similar to the distribution seen in an unselected population; however, has more cases in the higher mitotic score categories.

**Fig. 1:**
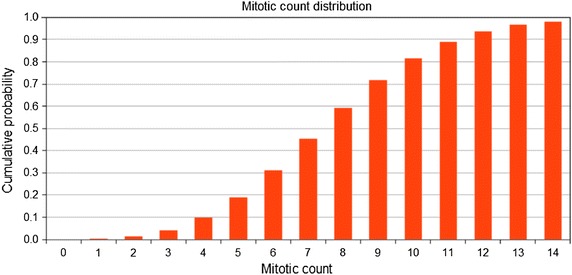
Cumulative probability (based on the binomial distribution) versus the mitotic count for a set of parameters (sample area = 2 mm^2^, cells/area = 2500 cells/mm^2^, true mitotic rate = 4.0 mitoses/mm^2^)

Then, in essence, the cumulative probability of each simulated mitotic count was calculated based on the knowledge of (1) the true mitotic rate per area, (2) the sample area, and (3) the cellular density. One such (sampled) mitotic count-probability distribution is seen in Fig. 1. The abscissa of Fig. 1 shows the (sampled) mitotic count (abscissa) versus the cumulative probability (ordinate).

The sampling processes, i.e. the simulated mitotic counts, were each represented by a random number between 0 and 1, which were considered equivalent to the percentile score of all possible sampling results. Thus, the random numbers could be substituted for the cumulative probability—in the (sampled) mitotic count–probability distributions (i.e. Fig. 1)—and thus converted to the sampled mitotic counts. The sampled mitotic counts were subsequently converted into (sampled) mitotic scores. The true and sampled mitotic scores were then determined based on the true and sampled mitotic densities, using the cutoffs of 3 and 8 mitoses/mm^2^ as in 2013 version of the CAP checklist [2], and the misclassification or agreement was tabulated. For each of the 1,000,000 cases the cellular density (2500 cells/mm^2^) was held constant.

For example:

If the random generated number is 0.42644 the mitotic score is determined to be 7, as the random number is less than the cumulative probability for 7 mitoses (0.45285) and greater than the cumulative probably for 6 mitoses (0.31318).

The number of specimens with a particular mitotic rate for the sample population are shown in Fig. 1, which was interpolated to generate a table of mitotic rates and their relative frequency in the population of specimens.

## Results

The percentage of agreement between the true mitotic rate and the sampled mitotic rate based on the three different sampled areas, 1.24, 3.74, and 5.00 mm^2^, are given in Tables 1, 2, and 3 respectively, for 1,000,000 simulated breast specimens. Some rounding and significant digits lead to totals not adding up to 100%. Additionally the accuracy, for each HPF area was also calculated. The misclassification rates are 16, 9, 8, 5, 4 and 4% for sample areas of 1.26, 3.74, 5, 10, 15 and 20 mm^2^ respectively (see Table 4). If one frames the comparison between the true mitotic score and the score generated by the simulated of mitotic count, as an inter-rater reliability problem, Cohen’s kappa is applicable as a measure. The kappa is 0.76, 0.87, 0.89, 0.92, 0.93 and 0.94 for sample areas of 1.26, 3.74, 5, 10, 15 and 20 mm^2^ respectively.

**Table 1:** Percentage classification for sample area 1.26 mm^2^

	True score 1 n = 655,196 (%)	True score 2 n = 245,122 (%)	True score 3 n = 99,682 (%)	Totals n = 1,000,000 (%)
Sample score 1	96	37	<1	72
Sample score 2	4	55	25	18
Sample score 3	<1	8	75	9

**Table 2:** Percentage classification for sample area 3.74 mm^2^

	True score 1 n = 682,735 (%)	True score 2 n = 209,385 (%)	True score 3 n = 107,880 (%)	Totals n = 1,000,000 (%)
Sample score 1	95	14	0	68
Sample score 2	5	82	18	22
Sample score 3	0	4	82	10

**Table 3:** Percentage classification for sample area 5.00 mm^2^

	True score 1 n = 684,813	True score 2 n = 212,349	True score 3 n = 102,838	Totals n = 1,000,000 (%)
Sample score 1	96	12	0	68
Sample score 2	4	83	13	22
Sample score 3	0	5	87	1

**Table 4:** Accuracy by area of all classifications of simulated breast specimens

Area (mm^2^)	Percentage correct	Percent misclassified
1.26	84	16
3.74	91	9
5.00	92	8
10.0	95	5
15.0	96	4
20.0	96	4

## Discussion

The three-tier system reproducibly separates score 1 and score 3; the lowest sample area (1.26 mm^2^) has less than 1% of cases misclassified as score 1 when it is truly score 3.

Intuitively, the middle group should have a higher misclassification rate than the other two, as it directly interfaces with the two other groups. The model reproduces this expected pattern; the middle group has the highest misclassification rate.

The smallest field diameter microscopes (0.40 mm), due to sampling error, incorrectly categorize an additional of 7% of all tumors when compared to the largest field diameter microscopes (0.69 mm), 27% more of cases in the mitotic score 2 group.

The results reproduce findings by Meyer et al.; the misclassification rate is quite high (9–16% of cases). There is a clear trend to less misclassification with greater sample areas and the misclassification rate has a non-linear relationship with the sample area where incremental increases in area have successively lower reductions in the misclassification rate, as shown by the flattening of the misclassification from 5 to 20 mm^2^ (Table 4).

**Fig. 2:**
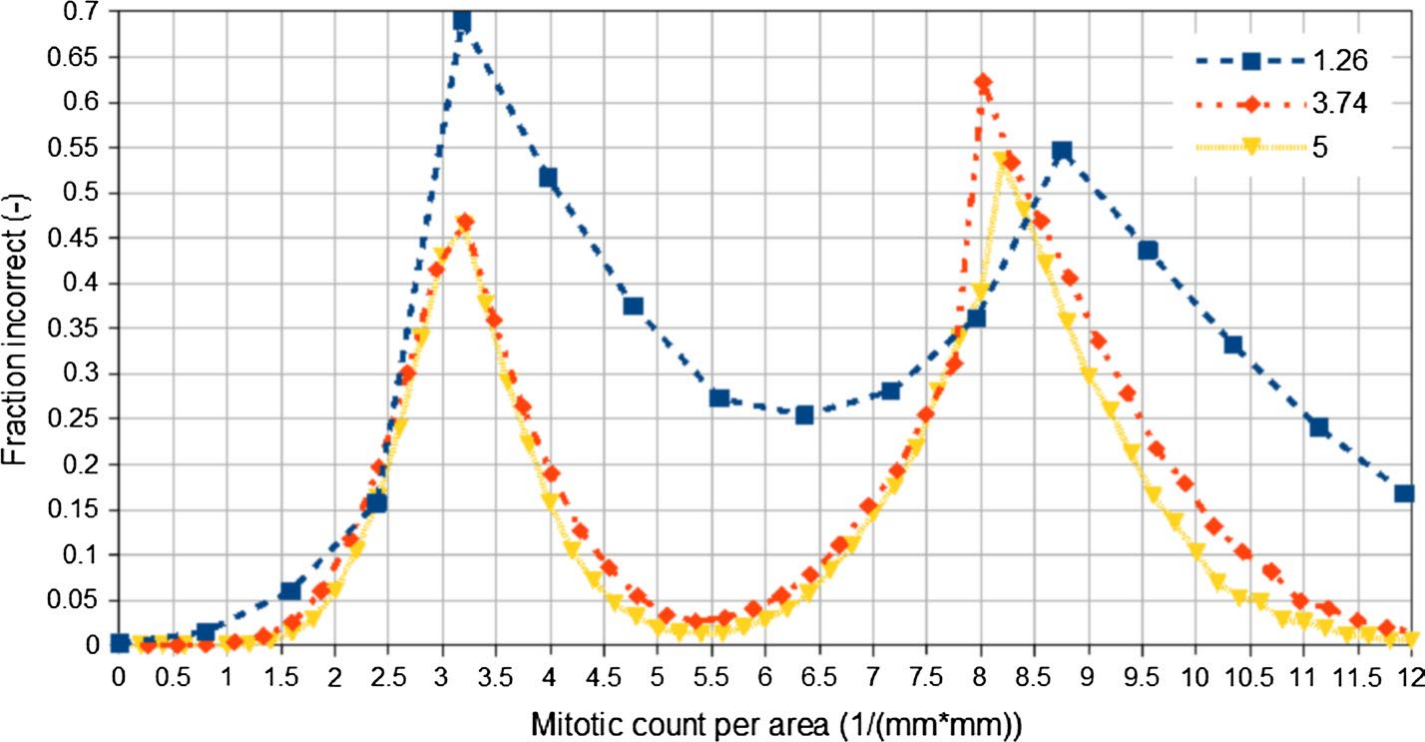
Missclassification versus true mitotic rate

The accuracy strong depends on the true mitotic rate, as a plot of the fraction incorrect versus the true mitotic rate demonstrates, Fig. 2.

## Limitations

The study did not consider the common practise of beginning the mitotic count with a mitotic figure. The effect of this practise could be calculated; however, it adds another level of complexity and likely does not change the overall conclusions. As well, the study did not systematically assess the impact of cellularity in the simulated breast specimens; however, some smaller calculations suggest it is not a significant factor (data not shown).

The findings are not corroborated by a large data set with patient outcomes and sample areas. This is a true short coming; however, we are not in possession of such a data set, though we hope this study will spurn some data mining by others. These findings regarding sampling theory as it applies to simulated breast specimens are practically self-evident, particularly if examined in the context of the vast experience with similar problems in opinion research (public polling) and manufacturing (statistical process control).

## Conclusions

Ten HPF is not a good standard sample area, as the misclassification rate is dependent on the microscope. The reproducibility of the mitotic score is poor, especially when close to the CAP Protocol cut-points of 3 and 8 mitoses/mm^2^ (see Fig. 2; Additional file 1: Appendix S1).

The mitotic count cut-points should be standardized and the sample area standardized; this could be accomplished by varying the number of HPFs counted and may be less complicated than the table in the CAP checklist (see Additional file 2: Appendix S2).

### Reducing misclassification

Generally, reducing the misclassification error requires a larger sample area, as noted by Meyer et al. [3]. However, we believe advocating larger sample areas (>5 mm^2^) would be impractical and needlessly tedious, as many cases can be assessed with a relatively small area. Also, as we have shown, a number of cases close to the cut-point, considered practically, will frequently be misclassified unless one samples the whole or at least the entirety of the most poorly differentiated component, of the tumour.

We believe a more rational approach would be to triage cases into (a) “needs a larger sample area”, and (b) “confident it is correctly classified”. The triage decision would be guided by a count on a (small) standardized sample area and a confidence interval around the cut-points. Cases deemed to need a larger sample area would be classified based on the larger sample area. We believe it is reasonable to draw the line after limited additional sampling; with a statement about the confidence interval—to make the clinician aware that a number of the cases will be misclassified by chance so that it can be taken into account. It is possible that some pathologists might already do such an activity, by performing repeated counts in several areas (Additional file 3).

We strongly believe that the term “high power field” and its cousins (“intermediate power field”, and “low power field”) should be completely abandoned as measures of area in pathology. Their continued use is offensive to any person that has given thought to why measures (such as the foot, millimeter and kilogram) are standardized or has some understanding of sampling theory.

Accurately quantifying proliferation will likely remain important for predicting cancer outcomes in the near term. Proliferative activity is used to subclassify breast cancer, has been quantified with Ki-67 labeling, and is central to commercial ancillary tests for breast cancer, e.g. Oncotype Dx [8].

The issue identified in this paper may explain, in part, why alternatives to the mitotic count have been sought; mitotic counts done by humans have limitations and have been done without much attention to sampling theory.

## Appendix

The following in an outline of the calculation. Words in quotes refer to the variable names in the program:

1. The expected number of mitoses (“m_expected”) is calcualted from the (randomly generated) true mitotic rate (“true_mitotic_rate”), using the sample area (“sample_area”).
2. The (binomial distribution) cumulative probability (“cdf_at_m_expected”) is calculated (using GNU Octave “binocdf” function) from the expected number of mitoses (“m_expected”), sample area (“sample_area”) and true mitotic rate divided by the cells per area (“TP”).
3. The number of mitoses counted (“m”) is then solved for by incrementally adding or subtracting from “m_expected” until the random sampling value (“P_sample”) is bounded by the (binomial distribution) cumulative probability of “m” and “m + 1” or “m” and “m-1”.
4. The mitotic count cut-points (“m_cp1” and “m_cp2”) are calculated from the mitotic rate cut-points (“cp1” and “cp2”) by, multiplying the mitotic rate cut-points by the sample area and rounding to the nearest mitosis.
5. The sampled mitotic count (“m”) is compared to the cut-points (“m_cp1” and “m_cp2”) to determine the sample mitotic score (“sample_m_score”).
6. The expected number of mitoses (“m_expected”) is compared to the cut-points (“m_cp1” and “m_cp2”) to determine the (true) mitotic score.

## Additional files

**Additional file 1: Table 1.** Compiled raw data for all areas. **Figure 1**. Fraction incorrectly classified by mitotic count/mm^2^ for total area of 1.26, 3.74, 5, 10, 15 and 20 mm^2^. **Figure 2**. Fraction incorrectly classified by mitotic count/mm^2^ for total area of 1.26, 3.74, 5, 10, and 15 mm^2^. **Figure 3**. Fraction incorrectly classified by mitotic count/mm^2^ for total area of 1.26, 3.74, 5, and 10 mm^2^. **Figure 4**. Fraction incorrectly classified by mitotic count/mm^2^ for total area of 1.26, 3.74, and 5 mm^2^. **Figure 5**. Fraction incorrectly classified by mitotic count/mm^2^ for total area of 1.26, 3.74, and 5 mm^2^. **Figure 6**. Fraction correctly classified by mitotic count/mm^2^ for total area 1.26, 3.74, 5 and 10 mm^2^. **Table 2**. Raw data generated for area of 1.26 mm^2^. **Table 3**. Raw data generated for area of 3.74 mm^2^. **Table 4**. Raw data generated for 5 mm^2^. **Table 5**. Raw data generated for 10 mm^2^. **Table 6**. Raw data generated for area 15 mm^2^. **Table 7**. Raw data generated for area of 20 mm^2^.

**Additional file 2: Table 1:** Recommended mitotic count points and fields to count. **Table 2**. Revised mitotic cut points. **Table 3**. confirmation of area equivalence. **Figure 1**. plot of variation of mitotic count per area as a function of field diameter.

**Additional file 3.** GNU octave computer code “get_sample_mitotic_score.m”.

**Additional file 4.** GNU octave computer code “mitotic_score_experiment_v7c.m”.

**Additional file 5.** GNU octave summary data “OUTPUT_mitotic_score_experiment_v7c.txt”.

## Abbreviations

CAP: College of American Pathologists
FD: Field diameter
HPF: High power field
